# PCSK5 mutation in a patient with the VACTERL association

**DOI:** 10.1186/s13104-015-1166-0

**Published:** 2015-06-09

**Authors:** Yukio Nakamura, Shingo Kikugawa, Shoji Seki, Masahiko Takahata, Norimasa Iwasaki, Hidetomi Terai, Mitsuhiro Matsubara, Fumio Fujioka, Hidehito Inagaki, Tatsuya Kobayashi, Tomoatsu Kimura, Hiroki Kurahashi, Hiroyuki Kato

**Affiliations:** Department of Orthopaedic Surgery, Shinshu University School of Medicine, Matsumoto, Japan; DNA Chip Research Inc., Kanagawa, Japan; Department of Orthopaedic Surgery, Faculty of Medicine, University of Toyama, Toyama, Japan; Department of Orthopaedic Surgery, Hokkaido University School of Medicine, Sapporo, Japan; Department of Orthopaedic Surgery, Osaka City University Graduate School of Medicine, Osaka, Japan; Department of Orthopaedic Surgery, Nagano Prefectural Children’s Hospital, Azumino, Japan; Division of Molecular Genetics, Institute for Comprehensive Medical Science, Fujita Health University, Toyoake, Japan; Endocrine Unit, Massachusetts General Hospital and Harvard Medical School, Boston, MA USA

**Keywords:** VACTERL association, *PCSK5* mutation, Whole exome sequencing

## Abstract

**Background:**

The VACTERL association is a typically sporadic, non-random collection of congenital anomalies that includes vertebral defects, anal atresia, cardiac defects, tracheoesophageal fistula with esophageal atresia, renal anomalies, and limb abnormalities. Although several chromosomal aberrations and gene muta tions have been reported as disease-causative, these findings have been sparsely replicated to date.

**Case presentation:**

In the present study, whole exome sequencing of a case with the VACTERL association uncovered a novel frameshift mutation in the *PCSK5* gene, which has been reported as one of the causative genes for the VACTERL association. Although this mutation appears potentially pathogenic in its functional aspects, it was also carried by the healthy father. Furthermore, a database survey revealed several other deleterious variants in the *PCSK5* gene in the general population.

**Conclusions:**

Further studies are necessary to clarify the etiological role of the *PCSK5* mutation in the VACTERL association.

**Electronic supplementary material:**

The online version of this article (doi:10.1186/s13104-015-1166-0) contains supplementary material, which is available to authorized users.

## Background

The VACTERL association (OMIM #192350) is a congenital disease characterized by the presence of at least three of the conditions of vertebral defects, anal atresia, cardiac defects, tracheoesophageal fistula, renal anomalies, and limb abnormalities. The VACTERL association occurs sporadically with a heterogeneous background, and its incidence is estimated at approximately 1 in 10,000 to 1 in 40,000 live-born infants [1]. The etiology of the VACTERL association is not still fully understood, although *HOXD13*, *ZIC3*, *PTEN*, *FANCB*, *FOXF1*, and *TRAP1* mutations have been reported as possible contributors to the VACTERL association or the VACTERL-like association as described previously [2–9]. Several microduplications and microdeletions are also reportedly associated with the VACTERL association [10, 11]. However, these findings have had sparsely be replicated.

Whole exome sequencing (WES) is a powerful tool in the investigation of genomic bases of human diseases. WES covers more than 95% of the exons of human genes, and 85% of the disease-causing mutations in Mendelian disorders are identified therein. In addition, many disease-associated single nucleotide polymorphisms (SNPs) are also located in this portion of the genome. Accordingly, WES is expected to provide information on nearly all functional, protein-coding regions in individuals with agnogenic congenital disorders [12, 13].

In this study, we analyzed a Japanese patient with the VACTERL association and his parents by WES and identified a novel frameshift mutation in the *PCSK5* gene. Since several non-synonymous variants in the cysteine-rich motif (CRM) of the *PCSK5* gene have been described in patients with the VACTERL association as a potentially responsible gene for this condition [14, 15], we further studied the causal role of the *PCSK5* mutation in the VACTERL association.

## Case presentation

A 10-year-old Japanese boy with the Fallot tetralogy (ventricular septal defect, branch pulmonary artery stenosis, right ventricular hypertrophy, and aortic overriding), congenital scoliosis affecting the eighth thoracic hemivertebra, unilateral renal anomaly of the right kidney, limb anomalies and dislocation of the right knee, and choroidal coloboma was referred to our institution. On the basis of four out of seven component features, the patient was diagnosed as having the VACTERL association by a board-certified pediatrician. The patient was born by vaginal delivery following a gestation period of 37 weeks and weighed 2,740 g at birth. He underwent heart surgery using a Rastelli procedure when he was 2 years of age. Since then, he has led a normal life apart from the abstinence of vigorous exercise. The parent’s family histories were unremarkable. There was no maternal or paternal history of congenital malformations, hypertension, diabetes, miscarriage, or consanguineous marriage.

## Methods

### Subjects

We analyzed the case of a Japanese boy with the VACTERL association based on the presence of four out of seven component features as determined by a board-certified pediatrician at our institution. After informed consent was obtained, peripheral blood samples were taken from the patient and his parents. Our study was approved by the Ethical Review Board for Human Genome Studies at Shinshu University School of Medicine.

### Exome capture and sequencing

A total of 3 μg of genomic DNA obtained from peripheral leukocytes of the patient and his parents was subjected to an exome capture procedure using the Agilent Sure Select Human All Exon Kit V5 (Agilent Technology, Tokyo) (target size: 50 megabases [Mb]) according to the manufacturer’s protocols. Briefly, genomic DNA was fragmented with Covaris (Covaris Inc., MA, USA) and purified using Agencourt AMPure XP beads (Beckman Coulter Inc., Tokyo). The quality of the fragmentation and purification was assessed with an Agilent 2100 Bioanalyzer. The fragment ends were repaired, an adenosine residue was added to the 3′ end of the fragments, and then SureSelect adaptors were ligated to the fragments using an Agilent SureSelect Library Prep Kit, ILM. At each step, the fragments were purified using Agencourt AMPure XP beads. The DNA libraries were amplified by PCR following quality confirmation. Exon-containing fragments were captured by biotinylated RNA library “baits” using an Agilent SureSelect Target Enrichment Kit. The captured DNA was purified with streptavidin-coated magnetic beads and re-amplified. The DNA libraries of the patient and his parents were sequenced using the illumina HiSeq system in 101-base-pair (bp) paired-end reads.

### Alignment and variant calling

Sequence reads were aligned to the human reference genome (GRCh37/hg19 + decoy sequences) obtained from the 1000 Genomes FTP site (ftp://1000genomes.ebi.ac.uk/) using the Burrows-Wheeler Aligner (BWA) version 0.6.2 [16]. Multiple identical reads from the exact same fragment were marked as duplicates and removed using Picard Tools version 1.8.3 (http://picard.sourceforge.net.). Exome target regions of the SureSelect V5 were downloaded from the Agilent SureDesign website (https://earray.chem.agilent.com/suredesign/) for calculation of read depth to assess coverage. Sequence data and coverage are shown in Additional file 1: Table S1. Local realignment around known indels, base quality score recalibration (BQSR), and variant calling using UnifiedGenotyper variant caller were performed with the Genome Analysis Toolkit (GATK) 2 version 2013.2 obtained from the Appistry website (http://www.appistry.com/gatk). Variant quality score recalibration (VQSR) using the GATK was performed to filter out false positive variants. This method builds a probabilistic model from a training set of known true mutations and assigns an accurate confidence score to each putative mutation call. The workflow of the GATK and its parameters were carried out as recommended by the GATK best practice guide (http://www.appistry.com/gatk).

### Additional WES Data for variant filtering and VQSR

We previously sequenced the genomes of 30 Japanese people, including 19 apparently healthy individuals. We included these data in our variant analysis since: (1) mutations found in the genomes of healthy individuals are not likely disease-causing and therefore are usable for further variant filtering; and (2) the VQSR of GATK requires at least 30 WES samples to achieve optimal results according to the GATK best practice guide. With these extra subjects, a total of 33 samples were available for variant analysis.

### Variant annotation, filtering, and family-based analysis

Functional annotations of the Ensembl database GRCh37.70 [17] and the possible effects of variants were added using SnpEff version 3.2 [18]. Using these annotations, VQSR-passing variants were filtered first for those that were predicted to alter amino acid sequences (missense, nonsense, and splice-site mutations and indels in coding regions), and then for those that were rare (<1.0% Minor Allele Frequencies [MAF] in the HapMap-JPT [Japanese in Tokyo, Japan] or the 1000 Genomes ASN [the East Asian population, composed mostly of Japanese and Chinese] databases). The filtering procedure of variants and their numbers are presented in Additional file 1: Table S1. Genetic mutations classified as de novo, dominant, or recessive (homozygotes or compound heterozygotes) were identified by trio-family-based analysis using an in-house script. The variants were also compared with the recently-released Human Genetic Variation Database (HGVD) (http://www.genome.med.kyoto-u.ac.jp/SnpDB/), which contained genetic variations determined by WES in 1,208 Japanese individuals.

### Variant validation

The variants identified via WES were confirmed using the Sanger method. Primer sequences were designed using Primer-BLAST [19]. PCR-amplified fragments were purified and then sequenced using an ABI3730xl DNA Analyzer (Life Technologies, Tokyo). Sequence data were analyzed with Sequence Scanner software version 1.0 (Life Technologies, Tokyo).

## Results

### Variant filtering, family-based analysis, and validation by Sanger sequencing

After the removal of variants that did not affect protein amino acid sequences or were common (≥1.0% MAF in HapMap-JPT or 1000 Genomes ASN databases) [20, 21], we analyzed the patient with the VACTERL association and his parents to identify genetically functional variants using the following classifications: (1) de novo mutations that were non-inherited or unique; (2) homozygous recessive mutations that were derived from both parents; and (3) compound heterozygous mutations for which the patient had at least two mutations derived separately from parents in the same gene. Two de novo mutations in two genes, one homozygous recessive mutation in one gene, and four compound heterozygote mutations in one gene in the patient were identified and validated by following Sanger sequencing (Additional file 1: Table S1). These mutations were not found in our in-house database of previously seqenced WES data obtained from 19 healthy Japanese individuals.

The identified two de novo mutations included a nonsense mutation in the NT5C3L (5′-nucleotidase, cytosolic III-like) gene and a missense mutation in the TTLL9 (tubulin tyrosine ligase-like family, member 9) gene. However, since both genes are functionally unknown, we could not conclusively determine their contribution to the VACTERL association.

### WES identified a novel frameshift mutation in the PCSK5 gene

**Figure 1:**
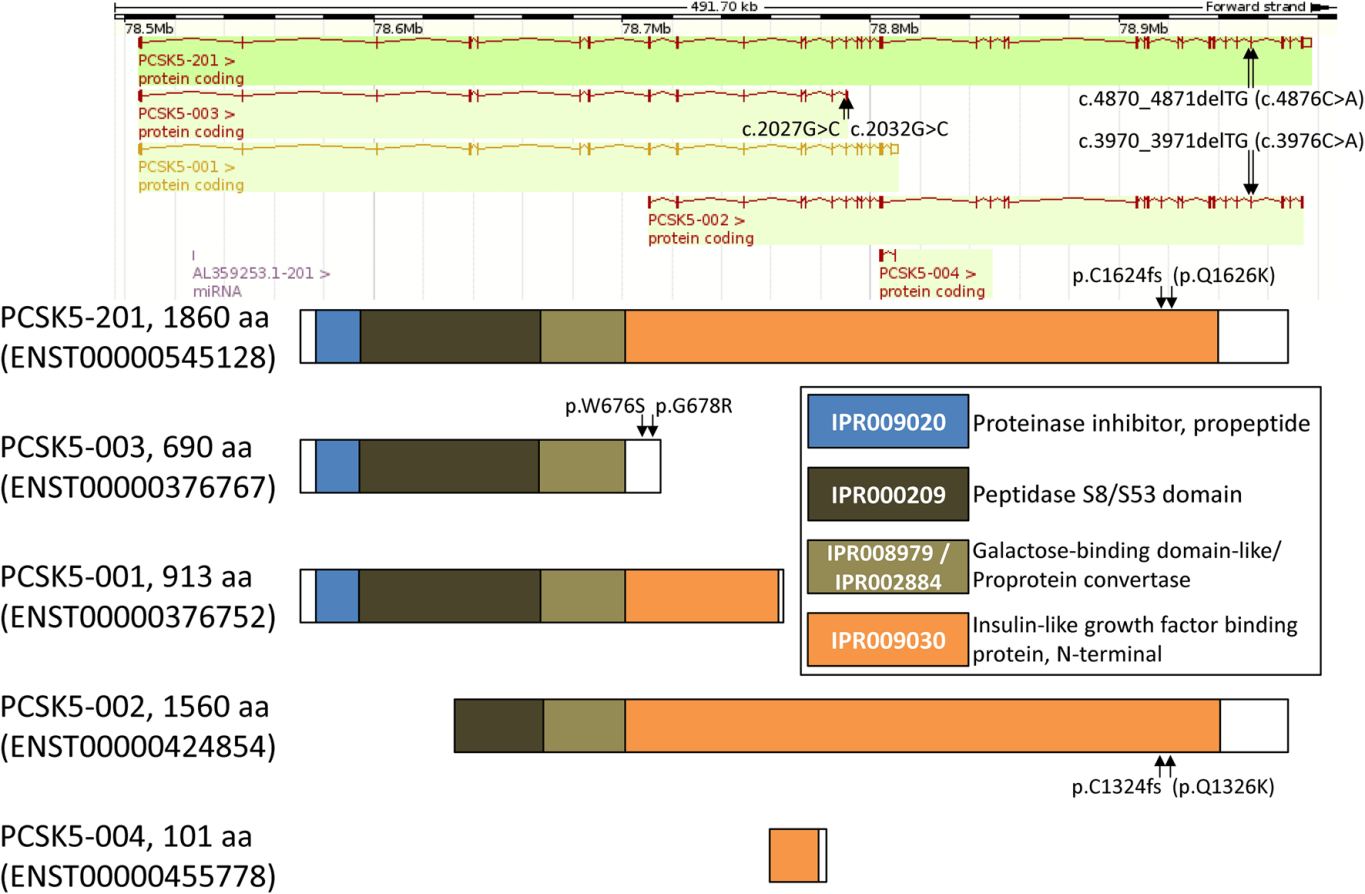
Exon and protein domain structures of the *PCSK5* gene. The transcripts and their exon structures are based on the Ensembl database (ENSG00000099139). Ensembl transcript IDs are shown in brackets. The protein domain structures are based on the InterPro database. InterPro domain IDs are shown in the legend with notes. The locations of the detected mutations are indicated by *black arrows*.

Among the candidates for recessive mutations, we focused on the *PCSK5* gene, which has been reported as one of the causative genes for the VACTERL association [9, 10]. We identified a novel frameshift mutation and three missense mutations as a compound heterozygote in the *PCSK5* gene in the patient that were validated by Sanger sequencing (Additional file 2: Table S2). The *PCSK5* gene has five transcript variants (splice variants/isoforms) according to the Ensembl database [17], and we called the mutations according to the longest transcript, PCSK5-201 (ENST00000545128). The frameshift mutation was c.4870_4871delTG (p.C1624fs) and a missense mutation was c.4876C>A (p.Q1626K) (Figure 1). The other two missense mutations only appeared in transcript PCSK5-003 (ENST00000376767) and we called them c.2027G>C (p.W676S) and c.2032G>C (p.G678R), respectively.

**Figure 2:**
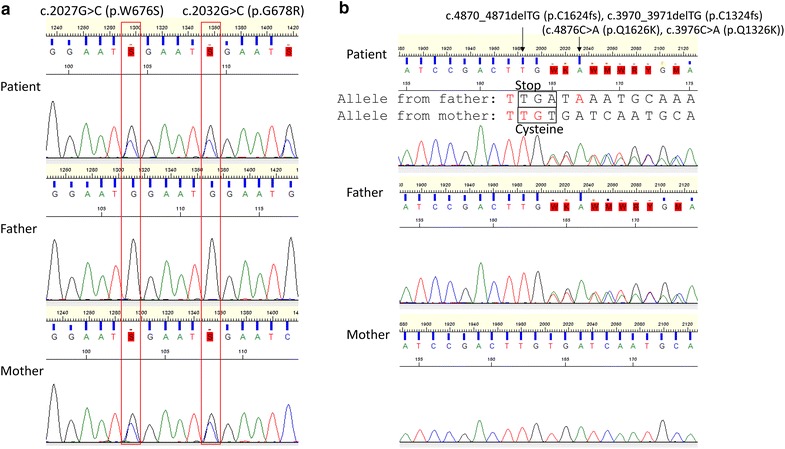
Novel *PCSK5* frameshift mutation in the patient as demonstrated by Sanger sequencing. **a** Two novel missense mutations are validated and show inheritance from the maternal allele. Note that another missense mutation just downstream of the two missense mutations is observed in the child, but not in the mother. The mutations are located in exon 14 of transcript PCSK5-003 (ENST00000376767). **b** A frameshift mutation is validated and shows inheritance from the paternal allele. The mutation is located in exon 34 of transcript PCSK5-201 (ENST00000545128) or exon 28 of transcript PCSK5-002 (ENST00000424854). Just after the frameshift, a cysteine codon is changed to a stop codon, which suggests a functional impact on this gene. Another missense mutation (c.4876C > A [p.Q1626K], c.3976C > A [p.Q1326K]) is validated in exon 34 but is presumed to be functionally irrelevant due to the upstream stop codon.

**Figure 3:**
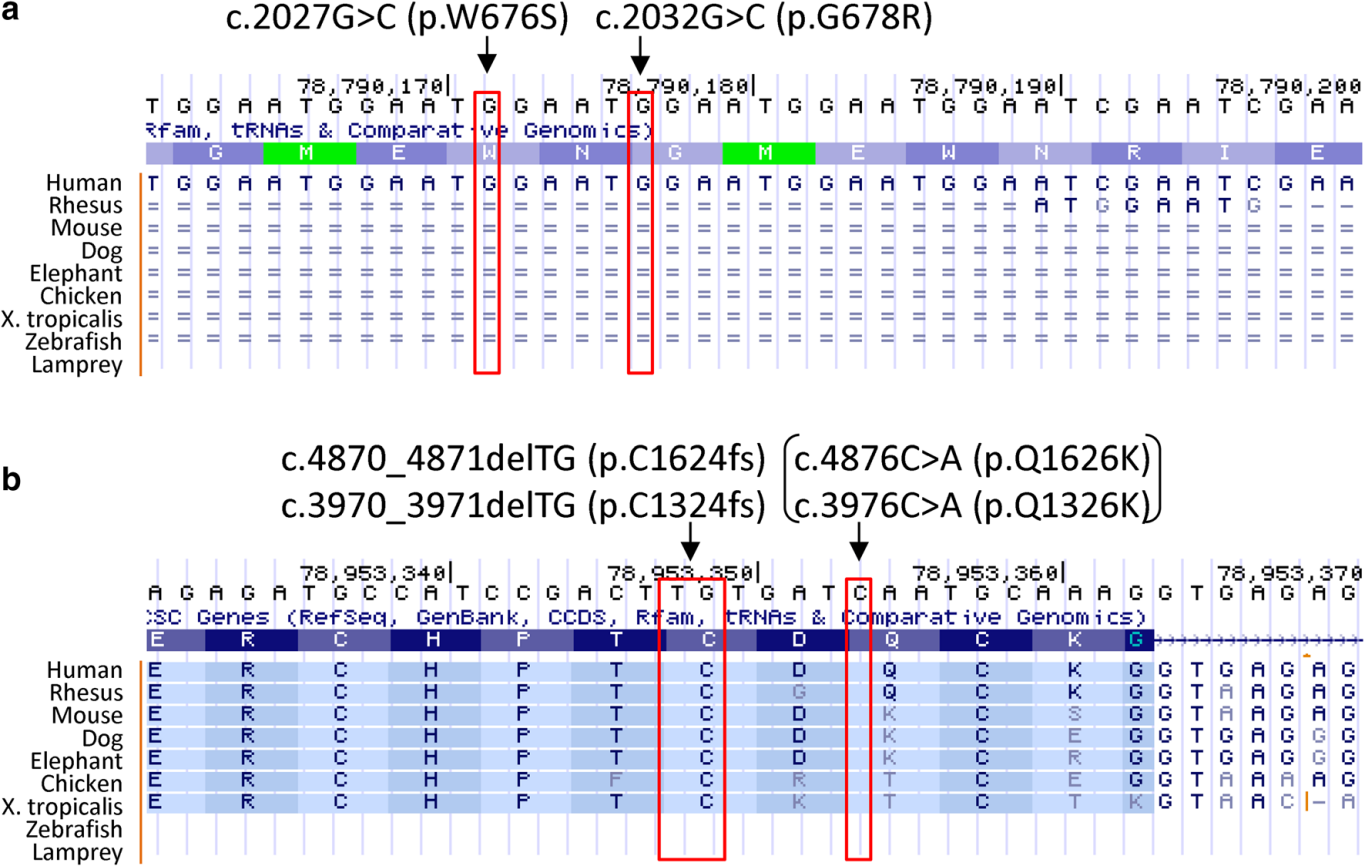
Evolutionary conservation of the region containing the frameshift mutation in the PCSK5 gene. **a** Two novel missense mutations in exon 14 of PCSK5-003 inherited from the maternal allele have no conservation across species. **b** The frameshift mutation in exon 34 (PCSK5-201)/28 (PCSK5-002) derived from the paternal allele affects highly conserved residues. The p.Q1626K (PCSK5-201)/p.Q1326K (PCSK5-002) mutation exhibits conservation among primates, but may be functionally irrelevant due to an upstream frameshift mutation.

Two missense mutations in exon 14 of the PCSK5-003 transcript (p.W676S and p.G678R) were also present in the patient’s mother. The genomic region of these elements was located within a short simple repeat of (GAATG)n (Chromosome 9: 78790113-78790255, 143 bp) according to the RepeatMasker of the UCSC genome browser (http://www.repeatmasker.org/). These missense mutations were found to occur due to repeat number variations of 5-nucleotide repeats since there were nucleotide variations in each repeat unit (Figure 2a). The 5-nucleotide repeats were transcribed only in the PCSK5-003 isoform. The evolutional conservation of the elements in which the mutations were detected was analyzed by multiple alignments using the UCSC genome browser [17], and this region demonstrated no conservation across species (Figure 3a). Taken together, these mutations are unlikely to affect the function of the gene product. Whereas the dbSNP database contains a single submission for these mutations (rs62556590, rs77249767) [20], those of HapMap-JPT, 1000 Genomes (including ASN) [21], and HGVD have no such entries.

A frameshift mutation and the other missense mutation were also present in the boy’s father (Figure 2b). Although the dbSNP and 1000 Genomes databases do not contain records of the frameshift or the missense mutations in exons 34 (PCSK5-201) or 28 (PCSK5-002), the HGVD has entries for 4 and 2 identical mutant alleles, respectively, among a total of 858 alleles (allele frequency: 0.5 and 0.2%, respectively). Both mutations were located in the PCSK5-201 and PCSK5-002 isoforms. The frameshift mutation results in a truncated protein that disrupts a region highly conserved across species (Figure 3b), suggesting that it might cause a functional alteration of PCSK5. The position of the missense mutation (p.Q1626K) may not be significant because it is located downstream of the frameshift mutation (Figure 2b).

PCSK5 proteins are composed of 3 or 4 domains according to predictions by the InterPro database version 46.0 [22] (Figure 1). The protein domain that included the frameshift mutation was that of an insulin-like growth factor binding protein (IPR009030) containing a cysteine-rich motif (CRM). CRMs are found in a variety of eukaryotic proteins that are involved in receptor tyrosine kinase signaling. CRMs are also considered to be responsible for interactions with cell surface heparan sulfate proteoglycans (HSPGs) via tissue inhibitors of metalloproteases (TIMPs) [5]. The frameshift mutation converts a cysteine residue to a stop codon to produce a truncated protein with the loss of the C-terminal half of the CRM domain or a loss of protein production due to nonsense-mediated mRNA decay.

Since we identified the p.C1624fs (PCSK5-201) frameshift mutation in the database for the general population (HGVD), we next searched for other possible deleterious mutations in the PCSK5 gene (Additional file 2: Table S2). We identified two nonsense mutations, c.3052C>T (p.Q1018X, rs373172614) and c.4615C>T (p.R1539X, rs140709872), in the *PCSK5* gene in the variant database from the general population. The frequency of these variants is 0.02%, which is several-fold higher than that of the VACTERL association. We also identified five frameshift mutations, c.1490_1491insACAC (p.N497fs rs138544337), c.1496_1497insC (p.R500fs, rs372197834), c.3915_3916insG (p.V1307fs, rs34898066), c.4665_4666insG (p.Y1557fs, rs34889598), and c.5095_5096insCC (p.D1699fs, rs138280866), in the general population. Altogether, it appears that the mutation in the *PCSK5* gene might be innocuous and not associated with the VACTERL association.

## Discussion and conclusion

PCSK5 is a proprotein convertase enzyme that cleaves prohormones at consensus sequences [23–25]. *PCSK5* is expressed in somites, the dorsal surface ectoderm, and primordial vertebral cartilage [23], as well as in the skeletal regions of the developing vertebrae, limbs, and craniofacium [24]. Recessive PCSK5 mutant mice induced by ethylnitrosourea exhibited hypoplastic hindlimbs, absent tail, cardiac malformations, palatal agenesis, tracheoesophageal malformation, pulmonary hypoplasia, exomphalos, and renal agenesis [15], and epiblast-specific *PCSK5* conditional knockout mice using Meox2Cre showed similar phenotypes [26]. These findings demonstrate that PCSK5 plays a pivotal role in skeletogenesis and organogenesis in mice. Meanwhile, non-synonymous *PCSK5* mutations were implicated in 4 of the 36 cases of the VACTERL association in humans reported by Szumska et al. and in 3 of the 39 cases described by Winberg et al. [14, 15].

It has also been reported that GDF11 is involved in murine skeletogenesis [27]. PCSK5 can cleave the GDF11 propeptide into its mature form [16], which suggests that PCSK5 activates GDF11 in mice. Mutant PCSK5 proteins were seen to not cleave the pro-protein of GDF11 [27]. In addition, *GDF11* knockout mice showed absent tail, palatal agenesis, renal agenesis, and increased numbers of thoracic vertebrae and ribs, all of which resembled the phenotypes of *PCSK5*-deficient mice [15]. Therefore, it is likely that the mechanism by which such phenotypes are observed in mice is via an inability to produce active GDF11 [15].

We witnessed a novel frameshift mutation in the *PCSK5* gene in our patient with the VACTERL association. Since three heterozygous missense mutations [14], four heterozygous missense mutations, and one homozygous missense mutation in the *PCSK5* gene [15] were previously reported in patients with the VACTERL association, we first thought this might be a strong candidate gene that was responsible for the patient’s phenotype. However, this is improbable for several reasons: (1) the healthy father also carries this mutation, (2) databases for the general population include this frameshift mutation, and (3) databases for the general population include not only this mutation, but also other deleterious mutations in the *PCSK5* gene, such as nonsense and frameshift mutations. Furthermore, the frequency of these variants is several-fold higher than that of the VACTERL association. A recessive trait was unlikely for the VACTERL association since there have been no previous reports on the simultaneous occurrence of this condition in siblings. Taken together, the *PCSK5* mutation appears to be benign and unrelated to the etiology of the VACTERL association.

It remains possible that the effect of the *PCSK5* frameshift mutation observed in this study might be dominant with incomplete penetrance. To date, Winberg and other groups have suggested that detected *PCSK5* variants could represent pathogenic entities with reduced penetrance [14, 15]. It is also possible that the maternally transmitted missense mutation, although innocuous alone, can affect phenotype when combined with the paternal frameshift mutation. Further mutations or copy number variations in other genes might have affected phenotype as well. Moreover, since the etiology of the VACTERL association is believed to be multifactorial, both modifier genes and environmental factors, such as hyperglycemia, may have precluded the development of this malformation [28].

In addition to the *PCSK5* mutations, we identified two de novo mutations in our patient. One was identified in the *NT5C3L* gene encoding a cytosolic nucleotidase-like protein, which might play a role in RNA metabolism. The other was harbored in the *TTLL9* gene encoding a ciliary protein that may act in tubulin glutamylation [29, 30]. Since ciliary genes are involved in some human malformation syndromes, they represent another candidate gene responsible for the patient’s phenotype. Further studies are necessary to clarify the elusive etiology of the VACTERL association.

## Additional files

**Additional file 1: Table S1.** CARE Checklist (2013) of information to include when writing a case report.
**Additional file 2: Table S2.** Summary of the mutations identified in the subjects and database.
